# Protists as determinants of the One Health framework

**DOI:** 10.1093/ismejo/wraf179

**Published:** 2025-08-22

**Authors:** Alejandro Berlinches de Gea, Julia Walochnik, Jens Boenigk, Kenneth Dumack, Fiona Henriquez, Sonja Rückert, Martin Simon, Stefan Geisen

**Affiliations:** Laboratory of Nematology, Wageningen University & Research, Droevendaalsesteeg 1, Wageningen 6708 PB, The Netherlands; Institute of Specific Prophylaxis and Tropical Medicine, Center for Pathophysiology, Infectiology and Immunology, Medical University Vienna, Kinderspitalgasse 15, 1090 Vienna, Austria; Department of Biodiversity, University of Duisburg-Essen, Universitätsstraße 5, 45117 Essen, Germany; Centre for Water and Environmental Research, University of Duisburg-Essen, Universitätsstraße 2, 45141Essen, Germany; Aquatic Ecosystem Analyses, Institute for Integrated Natural Sciences, Universitätsstraße 1, 56072 Koblenz, Germany; Department of Civil and Environmental Engineering, University of Strathclyde, James Weir Building, Montrose St, Glasgow G1 1XQ, United Kingdom; Centre for Water and Environmental Research, University of Duisburg-Essen, Universitätsstraße 2, 45141Essen, Germany; Department of Eukaryotic Microbiology, University of Duisburg-Essen, Universitätsstraße 5, 45141Essen, Germany; Molecular Cell Biology and Microbiology, Faculty of Mathematics and Natural Sciences, University of Wuppertal, Gaußstraße 20, 42119 Wuppertal, Germany; Laboratory of Nematology, Wageningen University & Research, Droevendaalsesteeg 1, Wageningen 6708 PB, The Netherlands

**Keywords:** protists, One Health, animals, humans, environment, pathogen, mutualism, element cycling

## Abstract

One Health connects three main health elements: humans, animals, and the environment. Protists influence all three, but their role in the overall One Health framework has been widely overlooked. Here, we highlight the key characteristics that make protists integral to the One Health framework and provide examples on the negative and positive effects of protists on each element. Most importantly, we emphasize how protists connect all One Health elements. Finally, we discuss how protists can be leveraged to enhance One Health. In conclusion, the vast diversity (phylogenetical, functional, and morphological) of protists is key in shaping One Health and can be targeted to improve individual One Health elements and their connections.

## Introduction

The “One Health” framework proposes tight links between the health of humans, animals, and the environment (including plants and microbes) [1]. The human microbiome has become an acknowledged driver of human health [2]. Also, the environmental microbiome shapes human health both directly, through causative agents or antagonists of human diseases, or indirectly, by influencing plant performance or ecosystem functions [3]. Typically, microbiomes are seen as the microbial community mainly consisting of bacteria and fungi, thereby largely ignoring the protists!

**Figure 1:**
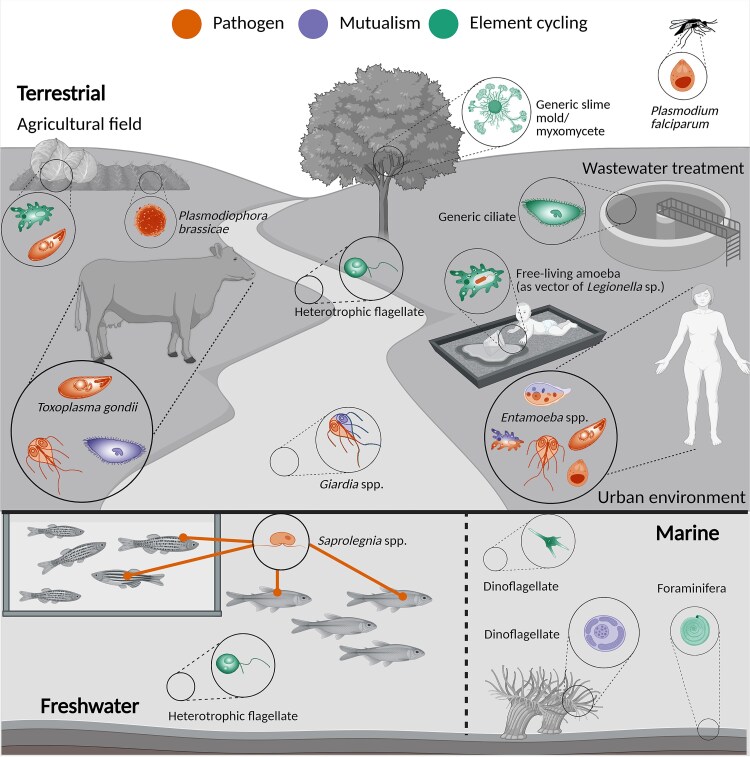
Visual representation of the different roles that protists play in One Health in terrestrial, freshwater, and marine ecosystems. The category “element cycling” embeds key processes such as primary production, decomposition, or nutrient regeneration. The icons solely represent the species or the protist group but not the specific stage in which they might be present in the environment (e.g. trophozoite, cyst). The presence of the same protist taxon in multiple hosts and ecosystems, performing distinct functions, illustrates their central role in One Health. Icons and connecting lines are differentiated by the three main functions protists perform:pathogenic activities, mutualism and roles in element cycling. Some icons are associated with more than one function, meaning that they might have different roles depending on the conditions. In the case of human symbionts, the mutualistic role is still under debate.

Protists are mostly single-celled organisms represented by potentially millions of species, distributed across the eukaryotic tree of life [4] and occurring in nearly all habitats (Fig. 1). Their phylogenetic and morphological diversity is mirrored by their vast functional diversity. These functions include the roles of protists as major consumers of bacteria, fungi, and other eukaryotes, as phototrophic carbon fixers, and as symbionts (from mutualists to parasites) of humans, animals, and plants [5] (Fig. 1). The role of protists in shaping the environment is evident in various geological formations. Large parts of the Alps are composed of limestone formed by foraminiferan protists; the famous cliffs of Dover mainly consist of sedimented calcifying haptophytes. The pyramids in Giza were built out of a limestone containing mainly the species *Nummulites gizehensis*, which also gave this foraminiferan its name. All animals, fungi, and plants evolved from protistan ancestors and at least one third of the human world population as well as most animal and plant species are colonized by protists. Many protists are pathogens, *Plasmodium falciparum*, the causative agent of malaria tropica, is responsible for >600 000 human deaths per year [6]; *Phytophthora infestans*, the cause of potato and tomato late blight, is among the most infamous threats to plant health [7]. Even though these protist pests are well known, most protists and their often-positive roles in One Health remain neglected.

**Figure 2:**
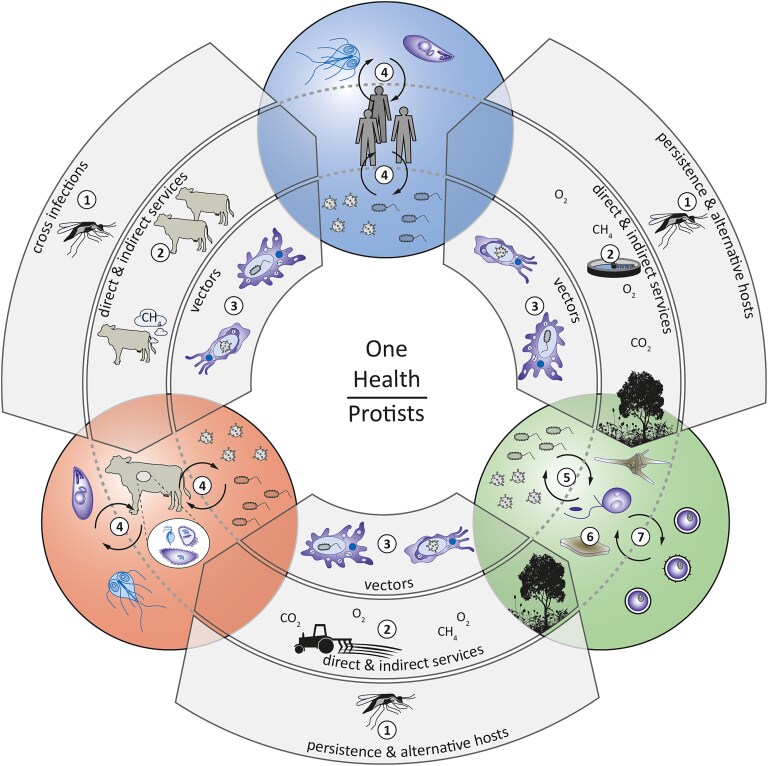
Representation of the roles protists play within the three individual One Health elements (human health: top, animal health: left, environmental health: right) and for the links between them. Numbers represent the following functions and applications: (1) cross infections/persistence and alternative hosts; (2) direct and indirect services among One Health elements (e.g. water purification, bioindication, biofertilizers, or food production); (3) vectors of pathogens; (4) direct effects on different elements of One Health such as pathogens or mutualistic protists; (5) pathogen removal and grazing; (6) primary production; and (7) algal blooms.

Here, we aim to provide a holistic overview of the immense impact of protists on virtually all One Health elements (Fig. 2). For that, we first focus on the unique aspects of protists in the One Health framework compared to other microorganisms. Then, we recap the role of protists as pathogens in the different One Health elements. To continue, we present their positive effects, including their complex roles in linking different One Health areas. Finally, we show possible applications of protists to improve specific components of One Health and how this ultimately affects the overall One Health framework.

## Key features: the importance of protists in the One Health framework

Protists are phylogenetically by far the most diverse eukaryotes [8]. Their species biodiversity is only matched by bacteria and viruses, and recent species have a morphological spectrum that spans at least six orders of magnitude in size [5]. As the last eukaryotic common ancestor (LECA) was already an organelle-bearing, highly complex protist [9], what is the reason for this complexity and evolutionary success?

In this section, we highlight three unique aspects of protist cell biology and genetics that distinguish them from other microbes and make them more comparable to metazoans, despite being mostly unicellular. The combination of these three aspects has greatly contributed to the evolutionary success and global distribution of protists, including the development of highly pathogenic life strategies, thereby constituting the basis of their significant impact on the One Health framework.

First, protist cells are in many ways different from those of prokaryotes and fungi, often they even resemble those of plants and animals. It can be argued that protist cells might often even be more complex than those of metazoans, as protists have subcellular structures and organelles with organ-like functions, without the association of different cell types and tissues as in metazoans. The phagotrophic vesicle cycle and osmoregulatory systems in protists can be compared to the intestine and kidney in animals and this vividly demonstrates that organ-like sub-functionalization of multicellular species can be realized by protists even in a single cell. These examples can be extended to cell walls, glycocalyx, extrusomes, and diverse motility structures like flagella, cilia, and pseudopodia.

Second, the ability for recombination, known as sex, as a key factor for genetic adaptation was certainly already present in the LECA and, therefore, exists in most protists [10]. Sex was a game changer in evolutionary history, allowing for the correction of deleterious mutations and increased genetic variability, thus supporting speciation [11]. Fungi, plants, and animals built upon the gain of sex as they originated from protist ancestors, making these “crown eukaryotes” appear as distinct tips of the protist-dominated eukaryotic iceberg.

The third factor concerns epigenetics, inherent to all protists to organize chromatin. Many protist species use complex epigenetic mechanisms, which paradoxically resemble metazoan differentiation processes. In metazoans, epigenetics enables cell differentiation of a single genome into different tissues, and this involves biochemical modifications of chromatin, histones, and DNA, to manifest transcriptional activity. Rather than simple transcriptional regulation of single genes, epigenetics allows concerted gene expression patterns resulting in precisely determined cellular phenotypes. Protists use epigenetics to achieve phenotypic plasticity of a single cell. Different life cycle stages, for instance, are the results of epigenetic differentiation processes and are essential for the complex transmission and infection strategies of parasitic protists, giving them access to new habitats or hosts, or the targeted use of vectors for dispersal. One of the best examples of epigenetically controlled life stages enabling a complex transmission and infection mechanism with several rounds of amplification is *Plasmodium*, with sexual reproduction in the mosquito and multiple asexual stages in the vertebrate host [12].

These different aspects highlight the considerable combination of features that is found exclusively in the protist kingdom: cellular complexity and diversity, genetic adaptation by sex, and the ability for epigenetic adaptation by advanced chromatin dynamics. Both adaptive aspects, in association with their unicellularity and r-selection strategy allow rapid adaptation and evolution, resulting in an incredibly diverse phylogenetic diversity. These complex cells have been adapted to nearly all ecosystems thus being important components of the One Health framework.

## Tiny menace: the dark side of protists in health and disease

The most prominent role of protists—both within individual health domains and in linking them through the One Health framework—is as pathogens that negatively impact the health of humans, animals, and plants. The diversity of pathogenic protists might lie in the millions of species [13] and is dominated by the group Apicomplexa, with over 300 currently described genera and, potentially, about one apicomplexan species infecting every animal species [14].

Several pathogenic protists exhibit high host specificity and have adapted to infect specific hosts and within these often specific organs or, if intracellular, specific cell types. Two examples for parasites specific for humans are *P. falciparum* and *Entamoeba histolytica*. The former causes malaria and infects red blood cells. Adult humans with 20–30 trillion erythrocytes can host several trillion cells of *P. falciparum* parasites, as over 30% of all erythrocytes can be infected [15]. Similarly, one human individual can harbor millions of cells of *E. histolytica* in their large intestine, and invasive amoebae may ingest more than a dozen erythrocytes per cell [16]. In animals, one of the most economically damaging diseases, costing several billion USD annually due to the loss of animals meant for food production, is avian coccidiosis, caused by *Eimeria* spp. Another economically damaging disease is saprolegniosis, caused mainly by protists of the genus *Saprolegnia*, which infect freshwater fish and pose a major challenge to fish production worldwide with losses up to 50% in certain regions [17]. Protists are also major pathogens of plants. A textbook example linking to One Health is *P. infestans*, the causative agent of potato blight, which gave rise to the Irish famine. This protist destroyed three quarters of the potato harvest after introduction to Ireland, reducing the Irish population by about half, through famine and emigration [18]. *Phytophthora infestans* is still a major threat to potato cultivation in the world, conservatively estimated to cost 6 billion USD per year [18]. Other economically relevant protist plant pests include *Plasmodiophora brassicae*, which causes clubroot disease, and *Phytomonas staheli*, causing coconut and oil palm wilts [19].

In contrast to all these more or less host-specific examples, some protists have broad host ranges and cross-species impacts, making control measures even more complicated. For example, *Trypanosoma cruzi*, which causes Chagas disease in humans, can infect various cell types across probably hundreds of animal species. *Toxoplasma gondii* is infecting about one-third of the total human population, and can infect virtually all warm-blooded animals [20].

Population dynamics of parasitic protists are generally linked to the population dynamics of their respective hosts as well as to the environmental conditions. Consequently, global changes, such as climate change, have direct and indirect effects on pathogenic protists. Moreover, human interference—including transportation, breeding or control of reservoir and vector host species, and treatment and prevention measures—plays a crucial role in determining population dynamics of parasitic protists. *Leishmania infantum*, causing visceral and cutaneous leishmaniasis in humans, is an example of a parasite that has been transported by humans around the globe [21]. This transportation has been predominantly within its main reservoir host, the dog, linking animal and human health. Moreover, the global dog population has been amplified to over 1 billion individuals by humans, thus enlarging the host population for this parasitic protist. Additionally, the vectors (species of sandflies) of *L*. *infantum* are longer active in many regions due to human-driven global warming, which leads to more transmission. Finally, the development of the pathogen within the sandflies is accelerated by higher temperatures. Anthropogenic activities may also increase the chance of pathogen spillovers, especially those from wildlife to humans. Examples are *Plasmodium knowlesi* spreading from monkeys to humans in Southeast Asia [22] or *Babesia microti* from rodents to humans in the Eastern USA [23]. However, parasitic protists can also spill over from humans to wildlife. Today in the USA, beavers act as important amplifiers of *Giardia* spp. of human origin after contamination of freshwater with human feces [24].

Major achievements toward prevention and control of many diseases that directly affect humans, livestock, or economically relevant plant species have been made in the past decades. However, pathogens that (also) infect wildlife are much more difficult to control, moreover, new diseases may emerge, often driven by human activities. One example is the emergence of amoebic gill disease caused by *Neoparamoeba perurans* with the surge of aquaculture worldwide [25], costing already millions of USD in losses [26]. Moreover, protists may act as reservoirs of other pathogens (prokaryotes, eukaryotes, and viruses) as well as of antibiotic-resistant bacteria and antibiotic-resistant genes [27], complicating the efforts to control disease in humans or animals. An example is the “Trojan Horse” case of *Acanthamoeba* spp., which can host and thereby protect a wide range of microorganisms from disinfection, such as *Legionella* spp. and other pathogenic bacteria [28].

## Tiny allies: exploring the positive impact of protists in ecosystems and health

Protists also play positive roles under the One Health framework as mutualists, primary producers, nutrient catalyzers, and regulators of microbial communities. In fact, protists can be keystone species by influencing microbiome composition and diversity and, thus, ecosystem functions such as determining plant performance [29].

The main positive direct link of protists with human health is as mutualists. Some protists are gut symbionts of humans, maintaining and shaping gut microbial communities and their functions, thus impacting the immune response and vulnerability to infectious and inflammatory diseases [30]. Examples are the parabasalids *Dientamoeba fragilis* and *Pentatrichomonas hominis*, or the amoebozoans *Entamoeba coli*, *Entamoeba hartmanii*, *Iodamoeba buetschlii*, and *Endolimax nana*. The presence of these gut protists is assumed to be positively related to gut microbial richness, which in turn is positively related to gut functions, but their prevalence is impacted by industrialization via food preferences, use of antibiotics, and lifestyle [30].

In ruminants, protist gut symbionts control microbial populations, synthesis of fatty acids, food degradation, rumen methanogenesis, and fiber digestion [31]. Some gut protists are obligatory mutualists, such as those living in the hindguts of termites, which degrade the lignocellulose from wood [32]. Protists also form symbiotic relationships in aquatic systems, where they enhance ecosystem stability and resilience. A textbook example of marine protist symbioses is the relationship of the dinoflagellate *Symbiodinium* and reef-building corals. The collapse of such relationships, as seen in coral bleaching due to anthropogenic-driven global changes, underscores the importance of these symbioses for maintaining biodiversity and ecosystem services in marine environments [33].

Mutualism is also part of the functional spectrum of protists in the environmental element linked to One Health. For instance, lichens, being associations between mostly autotrophic protists and fungi, are paradigmatic examples of mutualism. These organisms are of utmost importance for carbon fixation, especially in ecosystems without or with scarce vegetation cover [34]. Photosynthesis overall is a key function of protists in the environment. Free-living autotrophic protists, including diatoms, dinoflagellates, and other phytoplankton, contribute up to 50% of the total carbon fixed on Earth [35]. Soil algae are estimated to fix ~3.6 Pg of total carbon per year [36]. However, the role of protists in the global carbon cycle extends beyond fixation; they also participate throughout the entire cycle as saprotrophs, phagotrophs, and lysotrophs [37]. Moreover, protists influence not only the carbon cycle but also the cycles of other elements, such as silica and phosphorus. Diatoms, for instance, utilize silica to build their cell walls (frustules), playing a role in biogeochemical cycling of silicon [38]. Furthermore, protists contribute to the decomposition of organic matter practically in all the environments they are present, by facilitating the recycling of carbon and other elements [35, 37]. These increases in nutrient element cycling occur predominantly through indirect pathways, with protists catalyzing microbial activity and shaping the structure and function of microbial communities. The enhanced cycling of carbon, nitrogen, phosphorus, and other elements can increase plant performance and productivity as reviewed in, e.g. [5, 39], enhancing ecosystem services like food or wood production. While modify bacterial communities by hosting unicellular organisms, including plant-beneficial ones, positively affecting plant performance [40].

Protists do not only positively influence the One Health elements individually but also the links between them. Symbiotic protists in the rumens might impact animal health, also affecting human health via food production [41]. Similarly, the positive effect of predatory protists on plant performance and production enhances both animal and human health [5]. Even pathogenic protists may have a beneficial effect in the environment as some pathogenic protists such as *Parvilucifera sinerae* can reduce the harmful effects of algal blooms by controlling their populations [42]. Furthermore, pathogenic protists can increase biodiversity by reducing populations of dominant species, thereby liberating niches for rarer species to occupy (e.g. plant diversity; [43]).

## Tiny tools for planetary health: harnessing protists for environmental solutions

The above highlighted ecological and functional diversity of protists that greatly influence One Health makes protists ideal candidates for application. The evolution of protists added a hitherto unknown level of complexity to organismic interactions that is phagocytosis and predation, thus shifting the evolutionary driving forces from a physiological to a morphological focus. Unlike bacteria or yeasts, which mainly use membrane transport to ingest nutrients, protists recognize, hunt, catch, and ingest individual prey species (such as bacteria or other protists). Although hunting is believed to have required increased intelligence and skills in human evolution, this example shows that predation can be specific and far from simple filtration.

Today, the action of protist predation in shaping microbial-mediated biodegradation is the base for successful wastewater treatments. Furthermore, their sensitivity to biotic and abiotic changes, often being higher than bacteria and fungi, renders protists useful as bioindicators of ecosystem health [44]. Protists have long been employed to assess the quality of both natural and artificial water bodies. For instance, in 1999, the Bavarian State Office for Environment introduced “The Microscopic Picture in Biological Wastewater Treatment,” as a resource for wastewater treatment plant operators [45]. This resource enables wastewater treatment plant (WWTP) operators to evaluate the physicochemical parameters of their bioreactors by determining the community composition of designated indicator species through microscopic techniques and thus is significantly faster than conventional chemical measurements [46]. Certain groups of testate amoebae, such as Arcellinida, have been also suggested as bioindicators of freshwater quality, being sensitive to disturbances [47]. Similarly, soil protists have been suggested as indicators of both soil health and plant performance, with certain groups, such as cercozoan protists, being positively correlated to plant yield [48].

Beyond bioindication of plant performance, their roles in nutrient cycling, releasing nutrients to the soil via the so-called microbial loop, make protists potential tools in agroecosystems as biofertilizers, with products already being in place [49]. Another potential application for protists in agriculture concerns biocontrol agents due to their potential predatory action over pathogens, and by changing microbiome community composition toward pathogen-antagonistic bacteria [49].

In biotechnology and medicine, the highly developed cell biology of protists is now increasingly being used. A common example is the highly developed glycosylation machinery of the endoplasmic reticulum and Golgi apparatus, which enables the heterologous expression and complex glycosylation of proteins—such as human proteins—crucial for their proper function. Several protists, such as the ciliate *Tetrahymena*, offer an individual combination of benefits for heterologous expression in biotechnology. Its highly complex glycosylation machinery, which is easy to transform, cheap, and fast in cultivation, is not met by mammalian, yeast, or bacterial expression systems, and therefore already in application [50]. The complex cell biology of protists has a long scientific tradition in the molecular sciences, and the two Nobel Prizes for telomerase and ribozymes are just the tip of the iceberg of groundbreaking discoveries utilizing protists.

## Concluding remarks

We have presented protists here as key players in One Health, affecting all its individual elements and connecting human, animal, and environmental health. From one point of view, protists are among the deadliest and economically most damaging organisms. In contrast, their functional importance in numerous ecosystem functions, especially in element cycling (e.g. C or N) via decomposition, predation, or photosynthesis presents them as key mutualists in all One Health elements. Notably, the indirect roles of protists in One Health are often overlooked but crucial to understand how protists interact with other microbes, e.g. by acting as vectors for pathogens or mutualists, or by shaping microbiome compositions. As research continues to uncover the complexities of protist diversity, their contributions to environmental health will likely gain greater recognition, offering insights into sustainable ecosystem management and conservation strategies and numerous new biotechnological applications.

Overall, we believe that protists play a key role in planetary health by influencing different ecosystem functions, including elemental cycling and microbiome compositions, serving as models for industry, and connecting the different elements of the One Health.

## Data Availability

Data sharing not applicable to this article as no datasets were generated or analyzed during the current study.

## References

[1] Zinsstag J, Schelling E, Waltner-Toews D. et al. From “one medicine” to “one health” and systemic approaches to health and well-being. *Prev Vet Med* 2011;101:148–56. 10.1016/j.prevetmed.2010.07.003 20832879 PMC3145159

[2] Relman DA . The human microbiome: ecosystem resilience and health. *Nutr Rev* 2012;70:S2–9. 10.1111/j.1753-4887.2012.00489.x 22861804 PMC3422777

[3] Banerjee S, van der Heijden MGA. Soil microbiomes and one health. *Nat Rev Microbiol* 2023;21:6–20. 10.1038/s41579-022-00779-w 35999468

[4] Adl SM, Simpson AG, Lane CE. et al. The revised classification of eukaryotes. *J Eukaryot Microbiol* 2012;59:429–514. 10.1111/j.1550-7408.2012.00644.x 23020233 PMC3483872

[5] Geisen S, Mitchell EAD, Adl S. et al. Soil protists: a fertile frontier in soil biology research. *FEMS Microbiol Rev* 2018;42:293–323. 10.1093/femsre/fuy006 29447350

[6] Cowman AF, Healer J, Marapana D. et al. Malaria: biology and disease. *Cell.* 2016;167:610–24. 10.1016/j.cell.2016.07.055 27768886

[7] Fry WE, Birch PRJ, Judelson HS. et al. Five reasons to consider *Phytophthora infestans* a reemerging pathogen. *Phytopathology.* 2015;105:966–81. 10.1094/phyto-01-15-0005-fi 25760519

[8] Coleman DC, Geisen S, Wall DH. Chapter 5—Soil fauna: occurrence, biodiversity, and roles in ecosystem function. In: Paul E.A., Frey S.D. (eds.), Soil Microbiology, Ecology and Biochemistry (Fifth Edition). Amsterdam: Elsevier, 131–59. Retreived from https://www.sciencedirect.com/science/article/pii/B9780128229415000053.

[9] Koumandou VL, Wickstead B, Ginger ML. et al. Molecular paleontology and complexity in the last eukaryotic common ancestor. *Crit Rev Biochem Mol Biol* 2013;48:373–96. 10.3109/10409238.2013.821444 23895660 PMC3791482

[10] Cavalier-smith T . Origins of the machinery of recombination and sex. *Heredity* 2002;88:125–41. 10.1038/sj.hdy.6800034 11932771

[11] Silva VSD, Machado CR. Sex in protists: a new perspective on the reproduction mechanisms of trypanosomatids. *Genet Mol Biol* 2022;45:e20220065. 10.1590/1678-4685-gmb-2022-0065 36218381 PMC9552303

[12] Serrano-Durán R, López-Farfán D, Gómez-Díaz E. Epigenetic and epitranscriptomic gene regulation in *Plasmodium falciparum* and how we can use it against malaria. *Genes (Basel)* 2022;13:1734. 10.3390/genes13101734 PMC9601349 36292619

[13] Anthony MA, Bender SF, van der Heijden MGA. Enumerating soil biodiversity. *Proc Natl Acad Sci USA* 2023;120:e2304663120. 10.1073/pnas.2304663120 37549278 PMC10437432

[14] Morrison DA . Evolution of the apicomplexa: where are we now? *Trends Parasitol* 2009;25:375–82. 10.1016/j.pt.2009.05.010 19635681

[15] Gerald N, Mahajan B, Kumar S. Mitosis in the human malaria parasite *Plasmodium falciparum*. *Eukaryot Cell* 2011;10:474–82. 10.1128/EC.00314-10 21317311 PMC3127633

[16] Lohia A . The cell cycle of *Entamoeba histolytica*. *Mol Cell Biochem* 2003;253:217–22. 10.1023/A:1026055631421 14619972

[17] van den Berg AH, McLaggan D, Diéguez-Uribeondo J. et al. The impact of the water moulds *Saprolegnia diclina* and *Saprolegnia parasitica* on natural ecosystems and the aquaculture industry. *Fungal Biol Rev* 2013;27:33–42. 10.1016/j.fbr.2013.05.001

[18] Goss EM, Tabima JF, Cooke DE. et al. The Irish potato famine pathogen *Phytophthora infestans* originated in Central Mexico rather than the Andes. *Proc Natl Acad Sci USA* 2014;111:8791–6. 10.1073/pnas.1401884111 24889615 PMC4066499

[19] Schwelm A, Badstöber J, Bulman S. et al. Not in your usual top 10: protists that infect plants and algae. *Mol Plant Pathol* 2018;19:1029–44. 10.1111/mpp.12580 29024322 PMC5772912

[20] Djurković-Djaković O, Dupouy-Camet J, Van der Giessen J. et al. Toxoplasmosis: overview from a one health perspective. *Food Waterborne Parasitol* 2019;15:e00054. 10.1016/j.fawpar.2019.e00054 32095624 PMC7034049

[21] Lukeš J, Mauricio IL, Schönian G. et al. Evolutionary and geographical history of the *Leishmania donovani* complex with a revision of current taxonomy. *Proc Natl Acad Sci USA* 2007;104:9375–80. 10.1073/pnas.0703678104 17517634 PMC1890502

[22] Tobin RJ, Harrison LE, Tully MK. et al. Updating estimates of *Plasmodium knowlesi* malaria risk in response to changing land use patterns across southeast Asia. *PLoS Negl Trop Dis* 2024;18:e0011570. 10.1371/journal.pntd.0011570 38252650 PMC10833542

[23] Westblade Lars F, Simon Matthew S, Mathison Blaine A. et al. *Babesia microti*: from mice to ticks to an increasing number of highly susceptible humans. *J Clin Microbiol* 2017;55:2903–12. 10.1128/jcm.00504-17 28747374 PMC5625376

[24] Thompson RCA . The zoonotic significance and molecular epidemiology of giardia and giardiasis. *Vet Parasitol* 2004;126:15–35. 10.1016/j.vetpar.2004.09.008 15567577

[25] Nowak BF, Archibald JM. Opportunistic but lethal: the mystery of paramoebae. *Trends Parasitol* 2018;34:404–19. 10.1016/j.pt.2018.01.004 29422444

[26] Shinn AP, Pratoomyot J, Bron JE. et al. Economic costs of protistan and metazoan parasites to global mariculture. *Parasitol.* 2015;142:196–270. 10.1017/S0031182014001437 25438750

[27] Conco-Biyela T, Malla MA, Olatunji Awolusi O. et al. Metagenomics insights into microbiome and antibiotic resistance genes from free living amoeba in chlorinated wastewater effluents. *Int J Hyg Environ Health* 2024;258:114345. 10.1016/j.ijheh.2024.114345 38471337

[28] Guimaraes AJ, Gomes KX, Cortines JR. et al. *Acanthamoeba* spp. As a universal host for pathogenic microorganisms: one bridge from environment to host virulence. *Microbiol Res* 2016;193:30–8. 10.1016/j.micres.2016.08.001 27825484

[29] Gao Z, Karlsson I, Geisen S. et al. Protists: puppet masters of the rhizosphere microbiome. *Trends Plant Sci* 2019;24:165–76. 10.1016/j.tplants.2018.10.011 30446306

[30] Gerrick ER, Howitt MR. The lost kingdom: commensal protists in the gut microbiota. *Trends Microbiol* 2025;33:603–18. 10.1016/j.tim.2025.01.008 39952813

[31] Newbold CJ, de la Fuente G, Belanche A. et al. The role of ciliate protozoa in the rumen. *Front Microbiol* 2015;6:1313. 10.3389/fmicb.2015.01313 PMC4659874 26635774

[32] Gile GH . Protist symbionts of termites: diversity, distribution, and coevolution. *Biol Rev* 2024;99:622–52. 10.1111/brv.13038 38105542

[33] Hoegh-Guldberg O, Mumby PJ, Hooten AJ. et al. Coral reefs under rapid climate change and ocean acidification. *Science.* 2007;318:1737–42. 10.1126/science.1152509 18079392

[34] Seppey CVW, Singer D, Dumack K. et al. Distribution patterns of soil microbial eukaryotes suggests widespread algivory by phagotrophic protists as an alternative pathway for nutrient cycling. *Soil Biol Biochem* 2017;112:68–76. 10.1016/j.soilbio.2017.05.002

[35] Falkowski PG . The ocean’s invisible forest. *Sci Am* 2002;287:54–61. 10.1038/scientificamerican0802-54 12140954

[36] Jassey VEJ, Walcker R, Kardol P. et al. Contribution of soil algae to the global carbon cycle. *New Phytol* 2022;234:64–76. 10.1111/nph.17950 35103312

[37] Maillard F, Klinghammer F, Jassey VEJ. et al. Hidden decomposers: revisiting saprotrophy among soil protists and its potential impact on carbon cycling. *Soil Biol Biochem* 2025;205:109786. 10.1016/j.soilbio.2025.109786

[38] Armbrust EV . The life of diatoms in the world's oceans. *Nature.* 2009;459:185–92. 10.1038/nature08057 19444204

[39] Bonkowski M . Protozoa and plant growth: the microbial loop in soil revisited. *New Phytol* 2004;162:617–31. 10.1111/j.1469-8137.2004.01066.x 33873756

[40] Triplett LR, Taerum SJ, Patel RR. Protists at the plant-bacterial interface: impacts and prospective applications. *Physiol Mol Plant Pathol* 2023;125:102011. 10.1016/j.pmpp.2023.102011

[41] Solomon R, Wein T, Levy B. et al. Protozoa populations are ecosystem engineers that shape prokaryotic community structure and function of the rumen microbial ecosystem. *ISME J* 2022;16:1187–97. 10.1038/s41396-021-01170-y 34887549 PMC8941083

[42] Alacid E, Reñé A, Garcés E. New insights into the parasitoid *Parvilucifera sinerae* life cycle: the development and kinetics of infection of a bloom-forming dinoflagellate host. *Protist.* 2015;166:677–99. 10.1016/j.protis.2015.09.001 26605683

[43] Bever JD, Mangan SA, Alexander HM. Maintenance of plant species diversity by pathogens. *Annu Rev Ecol Evol Syst* 2015;46:305–25. 10.1146/annurev-ecolsys-112414-054306

[44] Zhao ZB, He JZ, Geisen S. et al. Protist communities are more sensitive to nitrogen fertilization than other microorganisms in diverse agricultural soils. *Microbiome.* 2019;7:33. 10.1186/s40168-019-0647-0 30813951 PMC6393985

[45] Pinther W, Ettl M, Hachenberg M. et al. Das mikroskopische bild bei der biologischen abwasserreinigung. Augsburg: Bayerisches Landesamt für Umwelt (LfU), 2022.

[46] Foissner W . Protists as bioindicators in activated sludge: identification, ecology and future needs. *Eur J Protistol* 2016;55:75–94. 10.1016/j.ejop.2016.02.004 27062305

[47] González-Miguéns R, Cano E, García-Gallo Pinto M. et al. The voice of the little giants: *Arcellinida testate* amoebae in environmental DNA-based bioindication, from taxonomy free to haplotypic level. *Mol Ecol Resour* 2024;24:e13999. 10.1111/1755-0998.13999 39044539

[48] Guo S, Xiong W, Hang X. et al. Protists as main indicators and determinants of plant performance. *Microbiome* 2021;9:64. 10.1186/s40168-021-01025-w 33743825 PMC7981826

[49] Hu S, Li G, Berlinches de Gea A. et al. Microbiome predators in changing soils. *Environ Microbiol* 2023;25:2057–67. 10.1111/1462-2920.16461 37438930

[50] Ruehle MD, Orias E, Pearson CG. Tetrahymena as a unicellular model eukaryote: genetic and genomic tools. *Genetics* 2016;203:649–65. 10.1534/genetics.114.169748 27270699 PMC4896184

